# Toxicity of various silver nanoparticles compared to silver ions in *Daphnia magna*

**DOI:** 10.1186/1477-3155-10-14

**Published:** 2012-04-02

**Authors:** Saba Asghari, Seyed Ali Johari, Ji Hyun Lee, Yong Seok Kim, Yong Bae Jeon, Hyun Jung Choi, Min Chaul Moon, Il Je Yu

**Affiliations:** 1 Young Researchers Club, Science and Research Branch, Islamic Azad University, Tehran, Iran; 2 Institute of Nanoproduct Safety Research, Hoseo University, Asan, Korea; 3 Institute of Nanoproduct Safety Research, Hoseo University, 165 Sechul-ri, Baebang-myun, Asan 336-795, Korea

**Keywords:** *Daphnia magna*, Silver, Nanoparticle, Colloid, Ion, Acute toxicity

## Abstract

**Background:**

To better understand the potential ecotoxicological impacts of silver nanoparticles released into freshwater environments, the *Daphnia magna *48-hour immobilization test was used.

**Methods:**

The toxicities of silver nitrate, two types of colloidal silver nanoparticles, and a suspension of silver nanoparticles were assessed and compared using standard OECD guidelines. Also, the swimming behavior and visible uptake of the nanoparticles by *Daphnia *were investigated and compared. The particle suspension and colloids used in the toxicity tests were well-characterized.

**Results:**

The results obtained from the exposure studies showed that the toxicity of all the silver species tested was dose and composition dependent. Plus, the silver nanoparticle powders subsequently suspended in the exposure water were much less toxic than the previously prepared silver nanoparticle colloids, whereas the colloidal silver nanoparticles and AgNO_3 _were almost similar in terms of mortality. The silver nanoparticles were ingested by the *Daphnia *and accumulated under the carapace, on the external body surface, and connected to the appendages. All the silver species in this study caused abnormal swimming by the *D. magna*.

**Conclusion:**

According to the present results, silver nanoparticles should be classified according to GHS (Globally Harmonized System of classification and labeling of chemicals) as "category acute 1" to *Daphnia *neonates, suggesting that the release of nanosilver into the environment should be carefully considered.

## Introduction

The increasing commercial application of engineered nanomaterials currently shows inventory listings of 1317 nanotechnology-based consumer products in 30 countries [1], plus the production of engineered nanoparticles is expected to reach approximately 60,000 tons in 2011 [2]. Silver is the most important nanomaterial mentioned in consumer product inventories, with 313 products (55.40% of all nano-products) [1], where the importance of nano-silver (nAg) is due to its antimicrobial properties [3-6] and application in the fields of material science, chemistry and physics. However, with the increasing presence of manufactured nanomaterials in consumer products, large quantities of nanoparticles could be released and eventually enter aquatic ecosystems, either wittingly or by chance [7-10], posing serious possible risks to the environment. Therefore, investigating the potential aquatic toxicity of nanomaterials has become an important issue.

*Daphnia magna*, a freshwater filter-feeding crustacean, is one of the most sensitive organisms used in ecotoxicity experiments and a standard test organism for the standard protocols of the U.S. Environmental Protection Agency (EPA), Organization for Economic Cooperation and Development (OECD), and International Standards Organization (ISO) [11,12]. Furthermore, since *D. magna *is at the bottom of the food chain in freshwater aquatic ecosystems, any change in its population quality or quantity can result in changes in the populations of other aquatic organisms. Even though the toxicity of AgNPs has already been studied in aquatic organisms such as Daphnia [13-16], the distinct characteristics of nanoparticles (e.g. preparation method, kind of capping agent, size, and shape) may change their effect on living organisms, leading some scientists to suggest that the toxicity of these new materials needs to be investigated case by case. Accordingly, the present study used *D. magna *as a model aquatic organism to evaluate the short-term toxicity of three different types of well-characterized silver nanoparticles, including two nano-Ag colloids, one of which has already been supplied as a reference material for the OECD WPMN (working party on manufactured nanomaterials) sponsorship programme on the testing of manufactured nanomaterials, and a nano-Ag powder suspended in water by sonication; moreover, an AgNO_3 _solution was also used to compare the toxicity effects of silver ions and silver nanoparticles. For each type of material, the mortality and immobilization rates, swimming type, and visible uptake of nanoparticles by *Daphnia *were investigated and compared.

## Materials and methods

### Nanoparticles and characterization

The present study used two types of colloidal silver nanoparticles and a suspension of silver nanoparticles as the sources of nanoparticles; plus, AgNO_3 _was used as the source of silver ions.

The first type of colloidal silver nanoparticles was donated by ABC Nanotech Co., LTD (Daejeon, Korea). The name of this blackish-brown product was SARPU 200 KW and, according to the information provided by the manufacturer, it was a water-based colloid containing 200000 mg/L spherical silver nanoparticles (5-25 nm). The purity of the silver nanoparticles was defined as 99.98%. The product was also extensively characterized and found to contain 20.48 wt% silver nanoparticles (thermogravimetry, TGA851, Mettler Toledo, Swiss) and 1.0 wt% citrate as the capping agent (HPLC, Waters 2690 analyzer) at pH 5.80. These silver nanoparticles have also been supplied as a reference material for the OECD WPMN sponsorship programme. Hereinafter, this colloid will be referred to as nAg1.

The second type of colloidal silver nanoparticles was purchased from Nano Nasb Pars Co., Ltd (Tehran, Iran). The name of this yellowish-brown product was Nanocid L2000 and, according to the information provided by the manufacturer, it was a water-based colloid containing 4000 mg/L spherical silver nanoparticles (average size 16.6 nm). The colloid product was synthesized using a novel process involving the photo-assisted reduction of Ag^+ ^to metallic nanoparticles, registered under United States Patent Application No: 20090013825. Briefly, 4.5 g of LABS (Linear alkyl benzene sulfonate) was dissolved in 95 ml of distilled water and then added to a solution containing 0.32 g of silver nitrate. After mixing thoroughly, 0.2 g of a hydrazine solution (0.03 M) was added, resulting in the formation of a yellowish silver colloidal solution. The measured pH of this product was 2.4, and some of its other properties were characterized in this study. Hereinafter, this colloid will be referred to as nAg2.

The powdered silver nanoparticles were purchased from Xuzhou Hongwu Nanometer Material Co., Ltd (Jiangsu, China). According to the information provided by the manufacturer, this black powder was 99% pure spherical silver nanoparticles with an average size of 20 nm. A stock suspension of 400 mg/L was prepared by dispersing 40 mg of this powder in 100 ml distilled deionized water, followed by vigorous vortexing (Thermo Scientific M37610) for 30 min at room temperature, then sonication for 6 hours in a bath-type sonicator (Branson 8510EXT-0011). Although this suspension was very stable after sonication, it was sonicated for a further 15 min immediately prior to each dosing. The pH of the final suspension was 7.32. Hereinafter, this suspension will be referred to as nAg3.

To compare the toxicity of the different silver nanoparticles with that of ionic silver, a stock solution of 400 mg/L AgNO_3 _(purity > 99.5%, Fluka chemika, Sigma-Aldrich, Switzerland) was prepared in distilled deionized water. The pH of the final solution was 6.43.

TEM analyses of nAg3 as a dry powder and in suspension, plus the nAg2 colloid were performed using an H-7100FA transmission electron microscope (Hitachi, Japan) with an acceleration voltage of 125 kV. In addition, TEM micrographs of nAg1 (FEI Tecnai G2-20-S-TWIN) were provided by ABC Nanotech Co., LTD (Daejeon, Korea). For each type of silver nanoparticle, the diameters of 700 randomly selected particles were measured at a magnification of 100,000 using Axio Vision digital image processing software (Release 4.8.2.0, Carl Zeiss Micro Imaging GmbH, Germany). EDX analyses of the dry powder, suspension, and colloids were also performed using an EX200 Energy-dispersive x-ray analyzer (Horiba, Japan).

Absorption spectral measurements were conducted on all the colloids and the suspension using a Spectra-MAX-PLUS 384 UV-visible spectrophotometer (Molecular Devices, USA) with a range of 190-1000 nm.

### *Daphnia *acute toxicity tests

The acute (48 h) toxicity tests were conducted in accordance with OECD guideline number 202 (*Daphnia *Sp. acute immobilization test) [12]. In this study, fully aerated M4 media were used as the exposure media and the test solutions were prepared immediately prior to use by diluting the different stocks mentioned above in the M4 media. After adding appropriate amounts of the stocks to the M4 media, the stock mixtures were stirred using a magnetic stirrer to distribute the suspension at as stable a concentration as possible. In addition, to avoid significant changes in the concentration of AgNPs during exposure, the media were refreshed every 24 h.

A series of preliminary experiments was conducted to determine the range of chemical concentrations that caused mortality in *D. magna*. According to the determined concentration ranges, effective concentrations were then selected for each substance (Table 1).

**Table 1:** Concentration gradients of different nanoparticles and AgNO_3 _used for acute toxicity tests (concentration ranges were selected according to preliminary experiments)

Chemical notation	Concentration (mg/L)									
nAg1 colloid	0.001	0.002	0.003	0.004	0.005	0.006	0.007	0.008	0.009	0.01

nAg2 colloid	0.001	0.0012	0.0015	0.0017	0.002	0.0022	0.0025	0.0027	0.003	0.0032

nAg3 suspension	0.1	0.125	0.150	0.175	0.2	0.225	0.25	0.275	0.3	0.32

AgNO_3 _solution	0.001	0.0012	0.0015	0.0018	0.0021	0.0024	0.0027	0.0029	0.0032	0.0034

Each test included a completely random design, consisting of ten treatments in triplicate and three control groups. Ten randomly selected neonates (younger than 24 h old) were placed in 100 ml exposure media in glass exposure beakers. All the tests were conducted in a water bath system with a constant temperature (20 ± 2°C) and 16 hr light/8 hr dark cycles. Since the presence of algae has been previously shown to affect the toxicity of nanoparticles [13] and the presence of organic matter shown to inhibit silver ion uptake by *Daphnia *[17], the animals were not fed during the experiments.

After 24 and 48 hours of exposure, the immobilization and mortality of the *Daphnia *in each test beaker were assessed using an Olympus CX41 microscope equipped with a digital camera (DIXI 3000 mega pixels, NEK Corp, Germany). According to Annex 1 of OECD 211, an animal was recorded as dead when it was immobile, i.e. not able to swim or no observed movement of appendages or the post-abdomen within 15 seconds after gentle agitation of the test container [18].

Furthermore, the live *Daphnia *were categorized in one of the following groups according to their swimming type: normal swimming (NOR), erratic swimming (ERR), *Daphnia *mainly at the bottom (BOT), and *Daphnia *mainly at the surface (SUR). Plus, any visible uptake and adsorption of nanomaterials by the *D. magna *were monitored and recorded.

### Statistical analysis

The 48-h EC10, EC50, and EC90 values, as well as their associated 95% confidence intervals (95% CI) were calculated using the US EPA Probit Analysis Program (version 1.5). In required cases, statistical analyses were carried out using standard ANOVA techniques, followed by Tukey's significant difference test (SPSS Ver. 17.0). Differences were statistically significant when p < 0.05. The particle size distributions of the three types of silver nanoparticle were statistically compared using a Mann-Whitney rank sum test.

## Results

### Particle characterization

**Figure 1:**
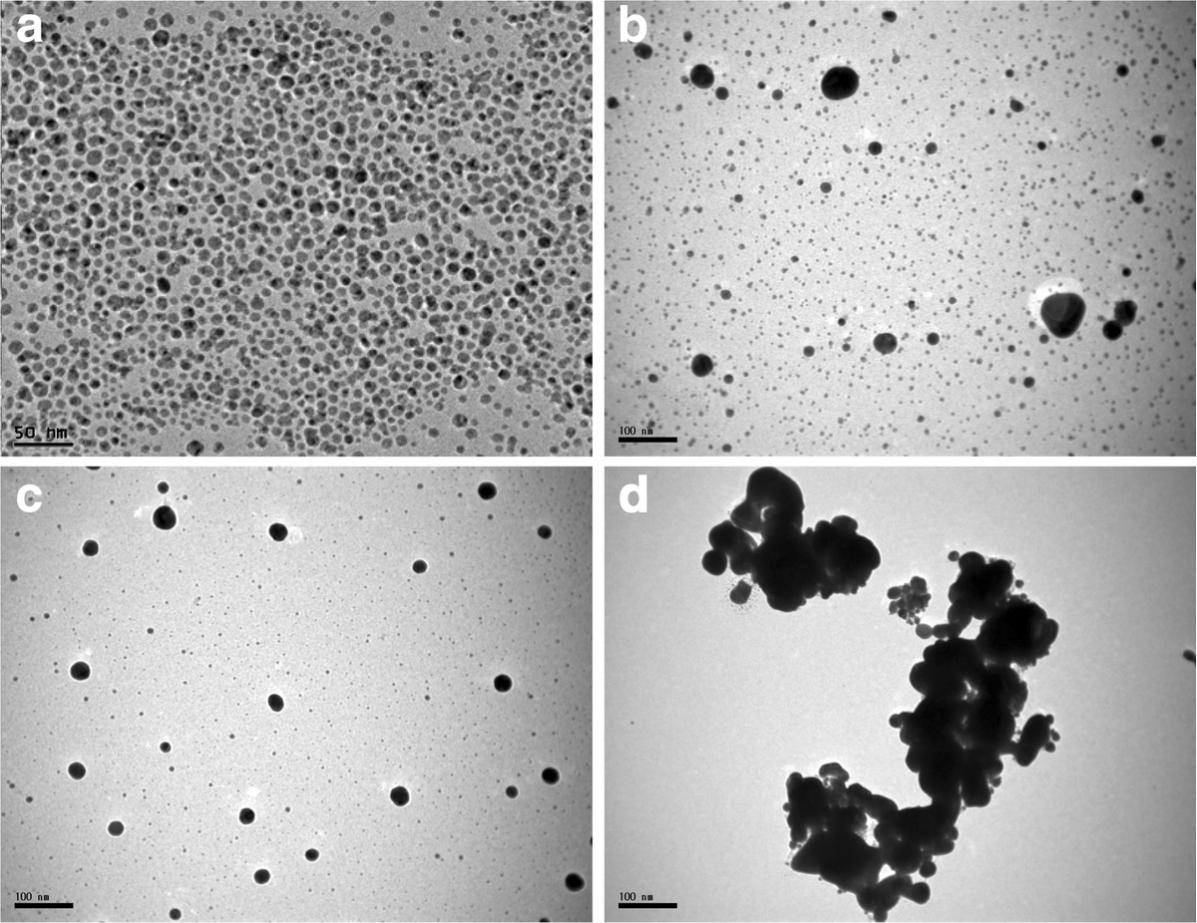
**TEM micrographs of different nanoparticles: (A) nAg1 colloid, (B) nAg2 colloid, (C) dry powder of nAg3, and (D) suspension of nAg3**.

**Figure 2:**
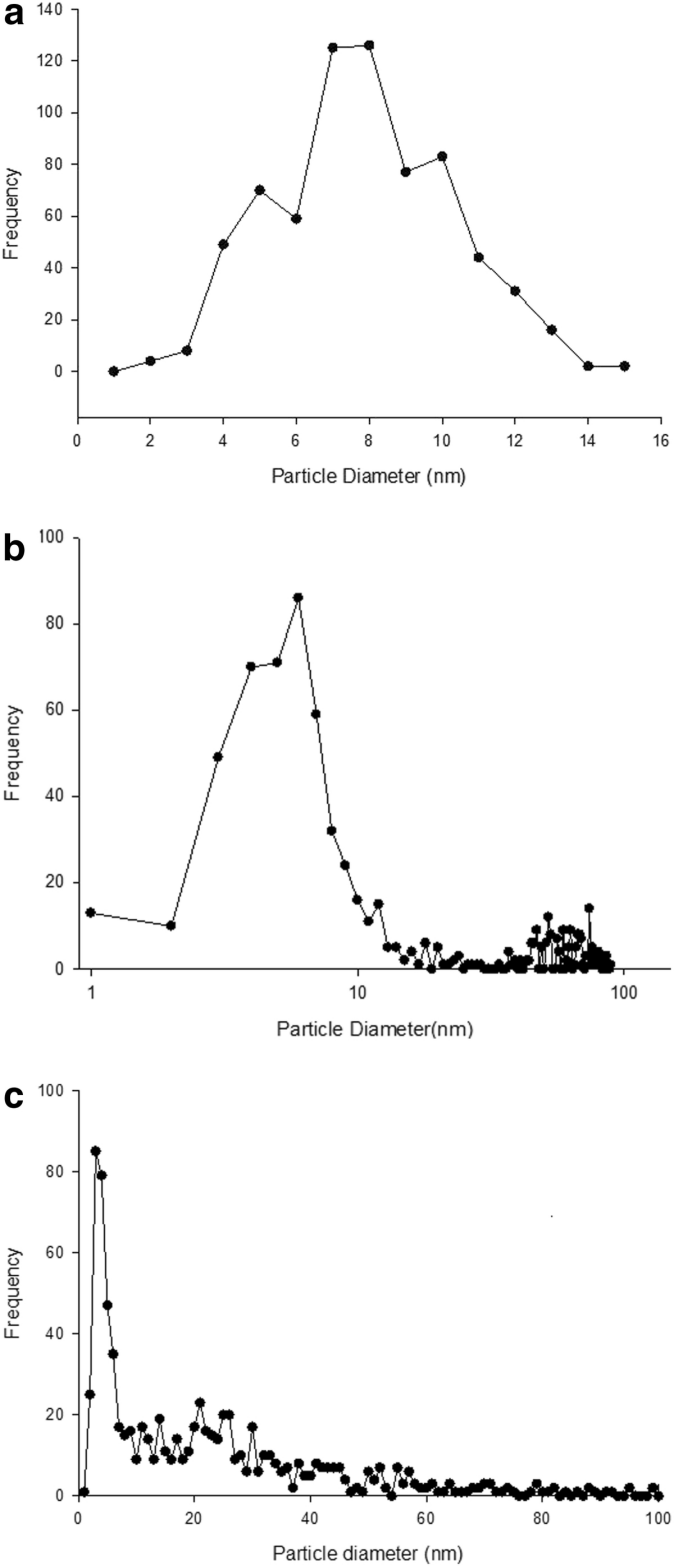
**Size distribution of particles based on number frequency determined from transmission electron microscope data in: (A) nAg1 colloid, (B) nAg2 colloid, (C) and dry powder of nAg3**.

**Figure 3:**
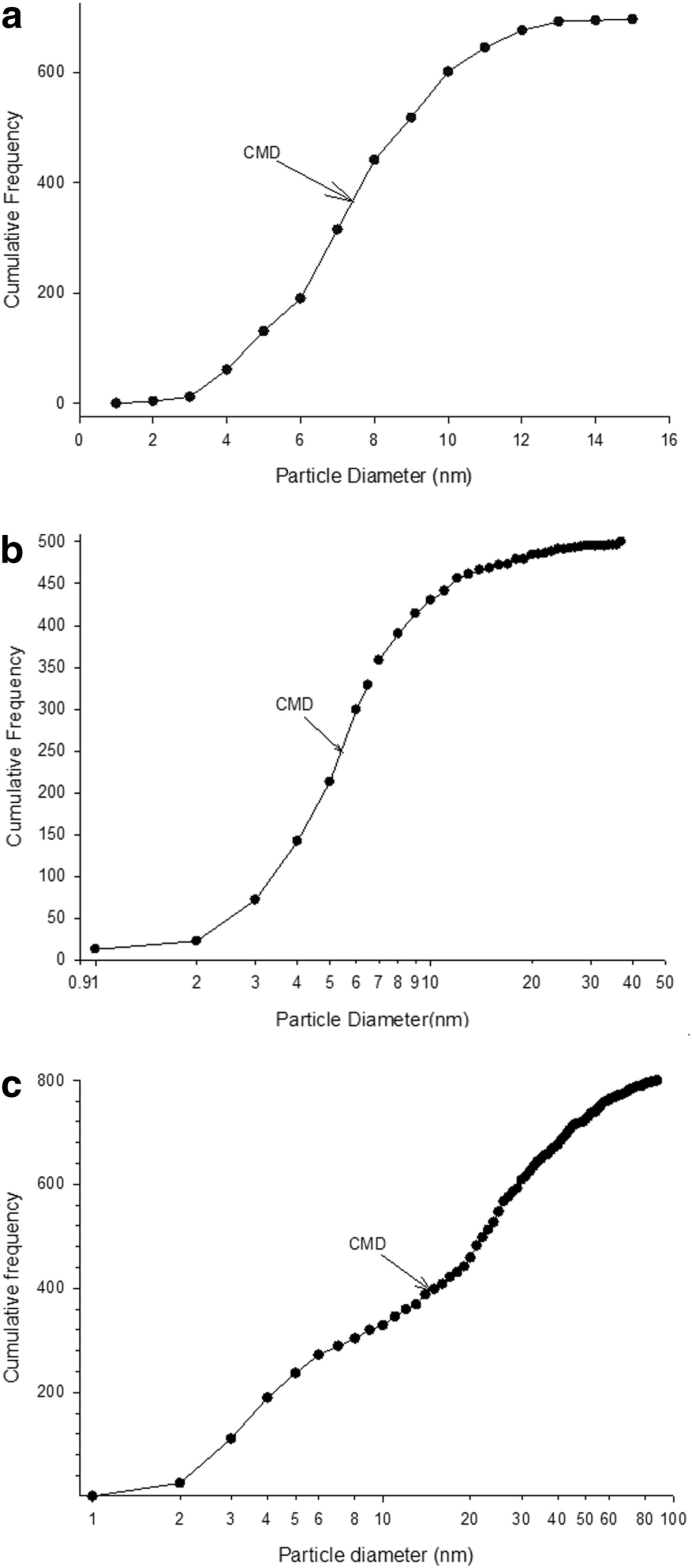
**Size distribution of particles based on cumulative frequency determined from transmission electron microscope data in: (A) nAg1 colloid, (B) nAg2 colloid, (C) and dry powder of nAg3**. Statistically significant differences were found among particle size distributions (*P *< 0.001, nAg1 vs nAg2; *P *< 0.001, nAg1 vs nAg3; *P *< 0.001, nAg2 vs nAg3).

In the nAg1 colloid observed by TEM, the particles were spherical in shape (Figure 1A), with a maximum diameter of 15.83 nm; 36.06% of the particles had diameters between 7 and 9 nm (Figure 2A); and the CMD (count median diameter) for the particles was 7.32 nm (Figure 3A). Also, the geometric mean diameter (GMD) and geometric standard deviation (GSD) of the colloidal silver nanoparticles were 7.96 nm and 1.35, respectively.

In the case of the nAg2 colloid observed by TEM, the particles were spherical in shape (Figure 1B), with a maximum diameter of 129 nm: 65.14% of the particles had diameters between 1 and 13 nm (Figure 2B), just 2.28% of the particles had diameters more than 100 nm, and the CMD for the particles was 6.47 nm (Figure 3B). Also, the geometric mean diameter (GMD) and geometric standard deviation (GSD) of the colloidal silver nanoparticles were 12.65 nm and 1.46, respectively.

In the case of the dry powder of nAg3 observed by TEM, the particles were spherical in shape (Figure 1C), with a maximum diameter of 161 nm: 85.97% of the particles had diameters between 1 and 45 nm (Figure 2C), just 1.34% of the particles had diameters more than 100 nm, and the CMD for the particles was 17.97 nm (Figure 3C). Also, the GMD and GSD of the dry powdered silver nanoparticles were 14.39 nm and 1.31, respectively. In the case of the nAg3 suspension, despite extensive sonication, the TEM images showed that in an aqueous environment about 52.9% of the nanoparticles were clumped together and formed large aggregates (Figure 1D). About 70.31% of the aggregates had diameters from 25 to 100 nm, while most of the others had diameters from 100 to about 250 nm.

The particle size distributions of the three types of silver nanoparticle were statistically compared using a Mann-Whitney rank sum test. Statistically significant differences were found among the particle size distributions (P < 0.001, nAg1 vs. nAg2; P < 0.001, nAg1 vs. nAg3; P < 0.001, nAg2 vs. nAg3). Thus, while the CMDs for nAg1 and nAg2 were similar, the distributions were different.

**Figure 4:**
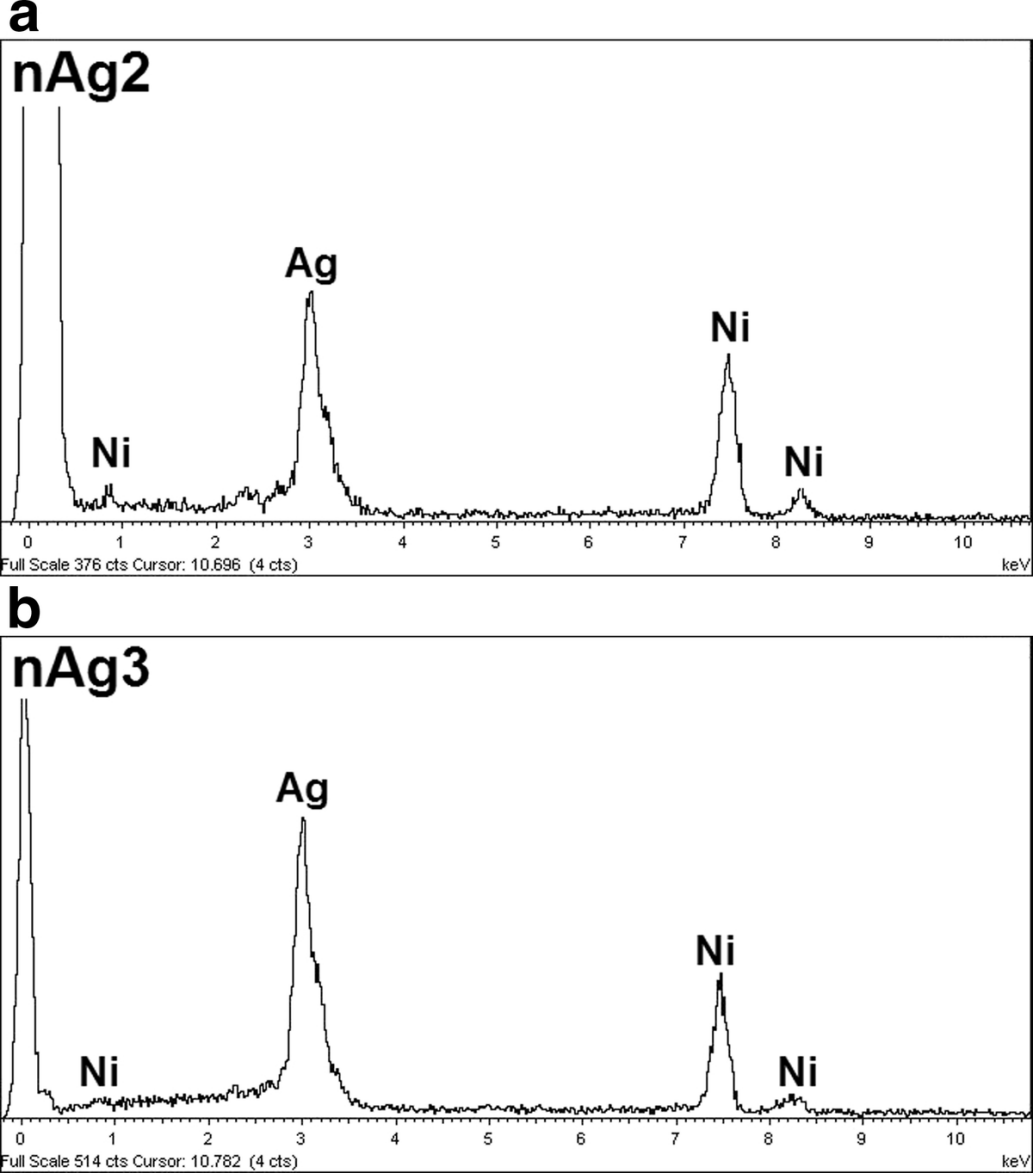
**EDX spectrometer patterns of nAg2 and nAg3; (Ni signals in EDX spectrometer are from TEM grid)**.

As seen in Figure 4, the EDX analyses revealed the presence of elemental silver in the nAg2 colloid and nAg3 preparation.

**Figure 5:**
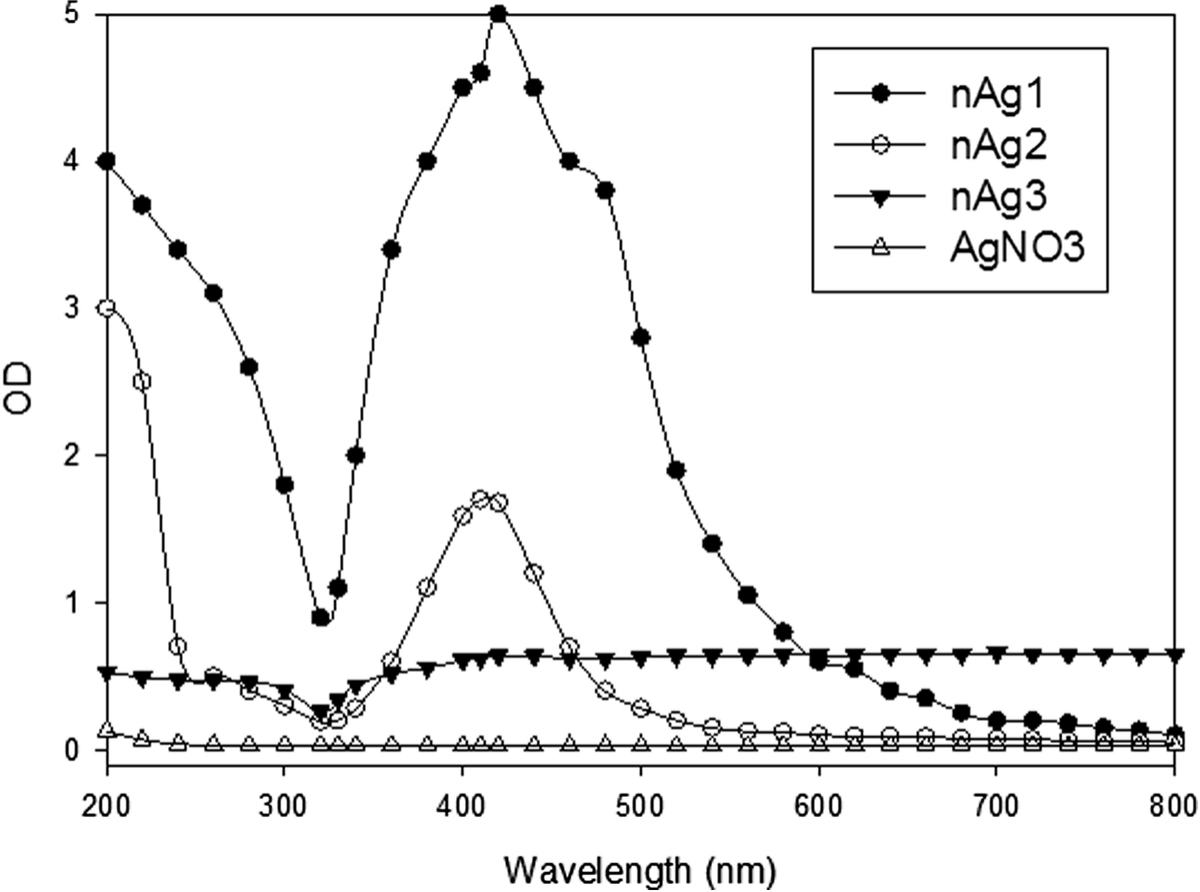
**UV-VIS absorption spectra for colloids of nAg1 and nAg2, nAg3 suspension, and AgNO_3 _solution**.

In the spectral scans of the nAg1 and nAg2 colloids, a strong surface plasmon resonance was centered at approximately 420 and 410 nm, respectively (Figure 5), which is similar to previous results for AgNPs [5,19-21]. However, for the nAg3 suspension, AgNO_3 _solution, and distilled water, no distinct peaks were observed (Figure 5). The observation of a strong surface plasmon peak has already been well documented for various metal nanoparticles, with sizes ranging from 2 to 100 nm [22,23]. In the case of nAg3, the increase in the particle size to more than 100 nm through aggregation may have been the reason for the lack of appearance of a distinct peak.

### Determination of effective concentrations (EC)

During the experiments, the mean and SD of the water pH and dissolved oxygen in the exposure vessels were 7.81 ± 0.13 and 7.44 ± 0.19 mg/L, respectively. Also, there was no significant difference between treatments in this regard (P > 0.05).

**Figure 6:**
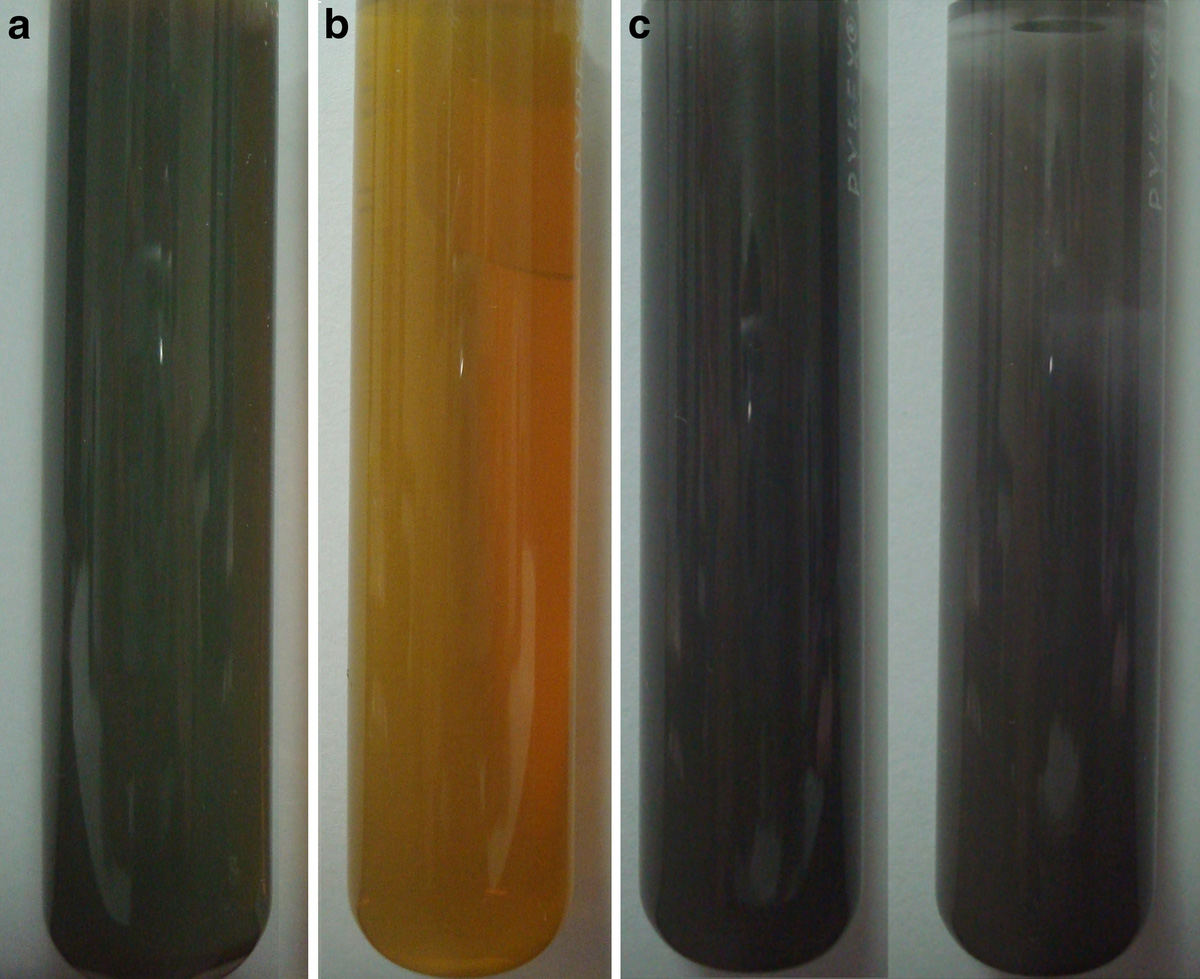
**Photograph comparing appearance of aqueous stocks of nanoparticles (400 mg/L) used for toxicity tests**. **A**: nAg1 colloid; **B**: nAg2 colloid; **C**: suspensions of nAg3 before (left) and after (right) 24 hours.

The nAg1 and nAg2 colloids (Figure 6) remained very stable in the exposure media (confirmed by UV-vis spectrophotometry, data not shown), and there were no signs of precipitation of the nanoparticles in the test beakers. In the case of the suspension of nAg3, sediments of aggregated nanoparticles became gradually visible at the bottom of the test beakers with the elapse of time; nonetheless, after 24 hours (and before refreshing the exposure media), most of the nAg3 particles were still suspended in the water (Figure 6).

During the exposure period, the mortality in the control groups was less than 5% for all the tests. The lowest concentrations of nAg1, nAg2, nAg3, and AgNO_3 _that caused 100% mortality of *Daphnia *after 48 hours were 0.006, 0.00325, 0.275, and 0.0032 mg/L, respectively. Also, the highest concentrations of nAg1, nAg2, nAg3, and AgNO_3 _that did not cause any mortality of *Daphnia *during 48 hours were 0.002, 0.001, 0.1, and 0.0015 mg/L, respectively. The average values of the effective concentrations and their 95% confidence limits are shown in Table 2. The median effective concentrations of nAg1, nAg2, nAg3, and AgNO_3 _were calculated as 0.004, 0.002, 0.187, and 0.0023 mg/L, respectively.

**Table 2:** Effective-concentration values, with lower and upper 95% confidence limits (CL), of different nanoparticles and AgNO_3 _for *Daphnia magna *neonates during 48 h

Chemical notation	Average EC10 (95%CL) (mg/L)	Average EC50 (95%CL) (mg/L)	Average EC90 (95%CL) (mg/L)
nAg1 colloid	0.003 (0.003-0.003)	0.004 (0.004-0.004)	0.005 (0.004-0.005)

nAg2 colloid	0.0015 (0.001-0.002)	0.002 (0.002-0.002)	0.003 (0.003-0.003)

nAg3 suspension	0.140 (0.110-0.158)	0.187 (0.165-0.205)	0.251 (0.226-0.301)

AgNO_3 _solution	0.0017 (0.0015-0.0018)	0.0023 (0.0022-0.0024)	0.0031 (0.0031-0.0037)

### Uptake and adsorption of NPs

**Figure 7:**
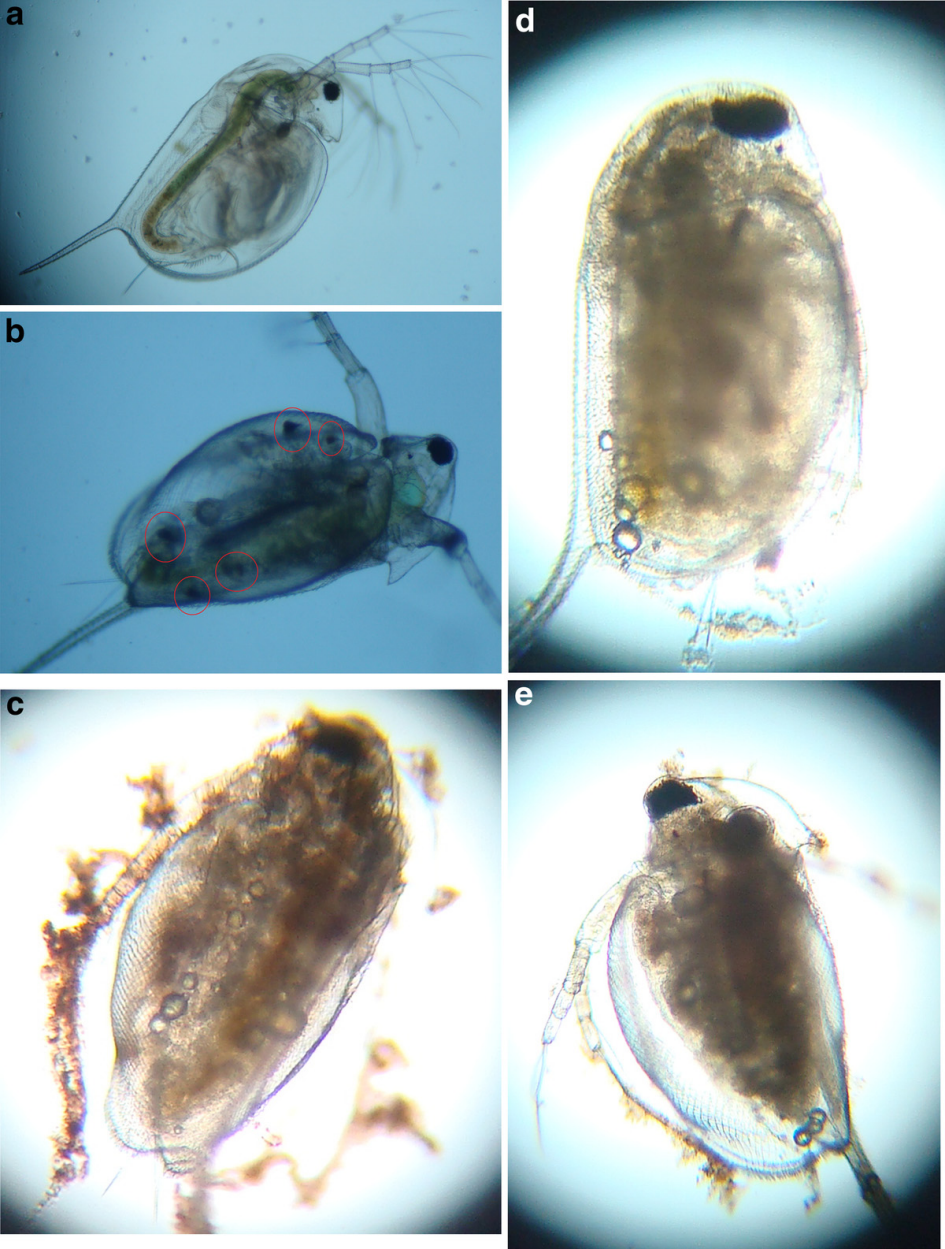
**Light microscope images of *daphnia *exposed to nAg1 and nAg2 colloids for 24 hours**. **A**: control; **B**: live *daphnia *exposed to 0.002 mg/L nAg1, pigmentation can been seen under the brood chamber (circles); **C**: dead *daphnia *exposed to 0.01 mg/L nAg2; **D**: live *daphnia *exposed to 0.004 mg/L nAg1; **E**: live *daphnia *exposed to 0.002 mg/L nAg2. In images C, D, and E, small bubbles can be seen under the carapace; plus, nanoparticle aggregates can be seen on the antennae, body surface, and also in the brood chamber.

**Figure 8:**
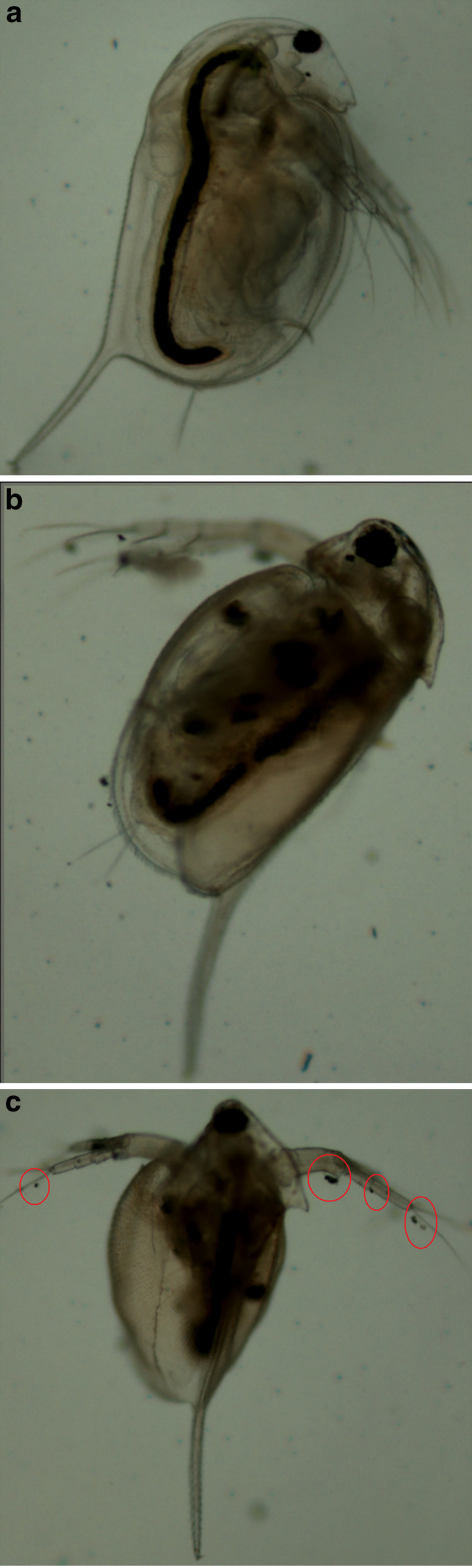
***D. magna *after 24-hour exposure to 0.15 mg/L aqueous suspension of nAg3**. **A**: black color of digestive tract shows uptake of nAg3. **B **and **C**: nanoparticle aggregates are attached to antennae and also seen in brood chamber.

After exposing the *Daphnia *to the nAg1 and nAg2 colloids and the nAg3 suspension, some pigmentation became visible in parts of the brood chamber that was not observed with the AgNO_3 _treatments and in the controls (Figures 7, 8); this pigmentation may have been a sign of nanoparticle accumulation under the carapace. In addition, at higher concentrations, nanoparticle aggregates were seen to be attached to the external body surface and appendages of the *D. magna *(Figures 7, 8), which in some cases affected the swimming ability. Also, a notable phenomenon with the colloidal treatments (nAg1 and nAg2) was the appearance of small bubbles under the carapace of the *Daphnia *(Figure 7).

With the nAg3 treatments, large amounts of a dark material were found in the gut tract of the *Daphnia *after nanoparticle exposure (Figure 8); thus, the nAg3 tested in this study was clearly ingested by the *D. magna*, resulting in accumulation in the gut. In some cases, the ingestion of the particles was enough to prevent the movement of the *Daphnia *through the water column and caused them to sink to the bottom of the beakers.

### Effects on swimming behavior

The normal and abnormal swimming of the live *Daphnia *are summarized in Table 3. In all the control groups, 100% of the live *Daphnia *exhibited completely normal swimming. In the case of abnormalities following exposure to the silver compounds, in the early stages, the *Daphnia *showed erratic swimming (ERR), while in the later stages, they migrated to the bottom (BOT) of the beaker or the water surface (SUR).

**Table 3:** Percentage of normal and abnormal *Daphnia magna *neonates during 48-hour exposure to different nanoparticles and AgNO_3_

Chemical notation	**Conc**.	%normal	%abnormal	%BOT	%SUR	% ERR
nAg1 colloid	0	100	0	0	0	0
	
	0.001	100	0	0	0	0
	
	0.002	0	100	0	0	100
	
	0.003	0	100	0	5.55	94.44
	
	0.004	0	100	9.9	0	90.09

nAg2 colloid	0	100	0	0	0	0
	
	0.001	73.34	26.66	26.66	0	0
	
	0.00125	58.63	41.37	34.48	6.89	0
	
	0.0015	55.56	44.44	30.7	13.74	0
	
	0.00175	48.7	51.3	43.61	7.69	0
	
	0.002	57.36	42.64	26.9	15.74	0
	
	0.00225	66.93	33.07	20.38	12.69	0
	
	0.0025	55.56	44.44	44.44	0	0
	
	0.00275	66.67	33.33	33.33	0	0

nAg3 suspension	0	100	0	0	0	0
	
	0.1	68.98	31.02	0	3.44	27.58
	
	0.125	71.43	28.56	17.85	10.71	0
	
	0.15	73.07	26.92	26.92	0	0
	
	0.175	73.69	26.3	15.78	10.52	0
	
	0.2	70	30	30	0	0
	
	0.225	73	27	27	0	0

AgNO_3 _solution	0	100	0	0	0	0
	
	0.001	93.34	6.66	6.66	0	0
	
	0.001275	86.67	13.33	6.66	6.66	0
	
	0.00155	86.67	13.33	13.33	0	0
	
	0.001825	78.27	21.73	17.39	4.34	0
	
	0.0021	54.46	45.54	22.77	22.77	0
	
	0.002375	57.15	42.85	42.85	0	0
	
	0.00265	0	100	70	30	0
	
	0.002925	0	100	90	10	0

In the nAg1 treatments, at concentrations up to 0.001 mg/L, 100% of the live *Daphnia *exhibited normal swimming; yet at higher concentrations (0.002 mg/L and more), all the *Daphnia *exhibited abnormal swimming. More than 90% of the abnormalities in the nAg1 groups were related to ERR.

About 26.6 to 51.3% of the live *Daphnia *in the nAg2 treatments exhibited abnormal swimming, yet the abnormalities were not dose dependent. Most of the abnormal *Daphnia *in order of frequency were BOT and SUR; and no ERR was observed in these groups.

In the case of the nAg3 treatments, about 26.3 to 31% of the live *Daphnia *exhibited abnormal swimming, yet the percentage of abnormalities did not differ significantly between the different concentrations (P > 0.05). In the lowest concentration (0.1 mg/L), most of the abnormal *Daphnia *were ERR, yet in the higher concentrations (0.125 mg/L and more), the abnormal *Daphnia *were BOT and SUR, respectively.

In the AgNO_3 _treatments, the percentage of abnormalities was dose dependent, and differed from 6.6% in the lowest concentration (0.001) up to 100% in the highest concentrations. Also, most of the abnormal *Daphnia *were BOT and SUR; and no ERR was observed in these groups.

## Discussion

The results of the present study demonstrated that silver nanoparticles are capable of causing acute toxicity in *D. magna*; however, the toxicity differed significantly according to the particle type. Several mechanisms have already been suggested to explain the toxic effect of silver nanoparticles; the presence of the nanoparticles themselves, the release of Ag^+ ^from nanoparticles, and the free radicals generated during dissolution in an AgNP suspension [24-28].

The 48-hour EC50 of the nAg3 suspension (nano silver powder) was determined to be 0.187 mg/L, which was relatively close to the results of Gaiser et al. [14] who previously reported that the EC50 of a sonicated nano-Ag suspension (588 nm average diameter of aggregates) was about 0.1 mg/L. Thus, the nAg3 was about 47 times less toxic than the nAg1 colloid and about 93 times less toxic than the nAg2 colloid. In previous literature on the toxicity of silver nanoparticles to *Daphnia *[13-16], the acute toxicity of laboratory-synthesized and ready-for-use silver nanoparticle suspensions (48-hour EC50 = 0.001- 0.003 mg/L) was higher than that of nano powders subsequently suspended in exposure water using sonication or agitation (48-hour EC50 = 0.031-0.1 mg/L). One possible explanation is that, in the case of nano powders that are subsequently suspended (as with nAg3 in the present study), despite the application of sonication, most of the particles tend to form aggregates and make large clumps in aqueous media, thereby decreasing the surface area to volume ratio and reducing the toxicity compared to the ready-for-use AgNP solutions. One exception to this is the study by Park and Choi [29], who showed that the lethal concentration of suspended powder AgNPs for *Daphnia *was about 0.001 to 0.002 mg/L, however, their study used a long sonication time (13 hours), followed by stirring for seven days and filtering through a 100 nm membrane; thus, a possible explanation for their high toxicity result was the dissolution of the silver ions into the aqueous media during the 7 days of stirring. In this regard, Kittler et al. [30] showed that the toxicity of AgNPs to human mesenchymal stem cells increased during storage due to the release of silver ions into the stock suspension.

The EC50 of the nAg2 colloid was determined to be 0.002 mg/L, which matched the results of Kennedy et al. [16], who reported that the EC50 of a 31 nm nano-Ag colloid (ASAP^®^) was 0.0018 mg/L. Meanwhile, the EC50 of the nAg1 colloid was determined to be 0.004 mg/L, so this colloid was two times less toxic than the nAg2 colloid. Even though the particle sizes of the nAg1 and nAg2 colloids were relatively similar (most particles were under 20 nm), the size distribution was significantly different. Thus, it is likely that other distinct characteristics of these two nano-Ag colloids were also related to the different toxicities, along with the particle size difference. For example, different coating agents may have led to the different toxicities. In this regard, Kennedy et al. [16] showed that the toxicities of various AgNPs with different coating agents (Citrate, EDTA, and Polyvinylpyrrolidone) were different (EC50 ranges were 0.0054-0.097 mg/L). Similarly, Zhao and Wang [31] showed that the EC50s of AgNPs with lactate, polyvinylpyrrolidone, and sodium dodecylbenzene sulfonate coatings were 0.0287, 0.002, and 0.0011 mg/L, respectively, for seven-day old *D. magna*. Also, Allen et al. [13] showed that the toxicity of uncoated particles was slightly higher than that of coated particles, and the toxicity of filtered suspensions was higher than that of unfiltered suspensions for *D. magna*.

The 48-hour EC50 for the silver ions (AgNO_3_) was determined to be 0.0023 mg/L, which matched well with previous literature [32-34]. Although, lower EC50s (0.0003, 0.0007, 0.0009, 0.0011, and 0.0016) have been reported in some other studies using *Daphnia *[13,16,31,35-37]. These differences in the toxicity thresholds may have been related to differences in the chemical purity, animal sensitivity, or test designs. In contrast, the EC50 value for nAg2 was relatively similar to that for AgNO_3_.

Overall, the comparative toxicity results for the different AgNPs and AgNO_3 _used in the current study suggest that silver nano powders subsequently suspended in exposing water are much less toxic than previously prepared nano Ag colloids, while colloidal AgNPs and silver nitrate are almost identical in terms of their toxicity.

Generally, EC50 data provides a good baseline for toxicity tests. According to GHS (Globally harmonized system of classification and labelling of chemicals, [38]) any substance with a 48 hr LC/EC50 (for *Daphnia*) of less than 1 mg/L must be classified as "category acute 1" to aquatic organisms. In addition, European Union legislation [39] and European Union Council Directive 67/548/EEC of 27 June 1967 [40] classified as "very toxic. Therefore, according to the present results, all the silver nanoparticle types and silver nitrate tested in the current study should be classified as "category acute 1".

Based on the present results, nAg3 was clearly ingested by the *D. magna*, resulting in accumulation in the gut. Thus, the results suggest that the aquatic exposure of aquatic organisms to such NPs could pose a risk of bioaccumulation, especially for filter-feeding copepods such as *D. magna*. In this regard, Zhao and Wang [31] showed that *Daphnia *can retain a large amount of AgNPs in their guts after ingestion. Other studies have also indicated that *D. magna *can uptake nanomaterials from test solutions [41-50]. Since *Daphnia *are part of the diet of other organisms, including fish, there is a potential for uptake and the subsequent transfer of nanoparticles to higher organisms.

Unlike the control groups that exhibited normal swimming, the *Daphnia *exposed to each type of silver compound showed at least one type of abnormality (ERR, BOT, or SUR). According to the results, it seemed that during the early stages of exposure, the *Daphnia *mostly showed erratic swimming, whereas in the later stages and in higher concentrations, they often migrated to the water surface or the bottom. In nAg1, the most common effect was ERR, while in nAg2, the *Daphnia *were mostly on the bottom or near the surface; so it seemed that nAg2 had a more severe effect on the *Daphnia *than nAg1. In the case of nAg3, which was more effective than nAg1 and nAg2 at higher concentrations, BOT was the most common abnormal behavior. In this regard, Strigul et al. [51] showed that *Daphnia *exposed to TiO_2 _nanoparticles were significantly slower after 24 h than the control *Daphnia*.

## Conclusion

This study investigated the acute toxicity of three types of silver nanoparticles and AgNO_3 _in *Daphnia magna*. The experimental results revealed that the different types of nanoparticles and silver ions produced distinct dose-dependent mortalities. In particular, the toxicity of the nano silver powder dispersed by sonication was lower than that of the colloidal silver nanoparticles. Therefore, the results suggested that the toxic responses were related more to the chemical characteristics and aggregation of the different nanoparticles. Future studies will investigate the chronic toxicity of different silver nanoparticles using *D. magna*.

## Competing interests

The authors declare that they have no competing interests.

## Authors' contributions

SA, SAJ, JHL, YSK, YBJ, HJC and MCM performed all necessary experiments, SA, SAJ, JHL and IJY analyzed data and wrote manuscript. All authors read and approved the final manuscript.
